# An Adolescent Patient With Hip Pain: Slipped Capital Femoral Epiphysis

**DOI:** 10.4021/jocmr503w

**Published:** 2011-04-04

**Authors:** Leyla Solduk, Ozgur Sogut, Halil Kaya, Mehmet T Gokdemir, Ugur Ozkanli

**Affiliations:** a Department of Emergency Medicine, Harran University, Faculty of Medicine, Sanliurfa, Turkey; b Department of Orthopedics and Traumatology, Harran University, Faculty of Medicine, Sanliurfa, Turkey

## Letter to the Editor:

**Figure 1.**
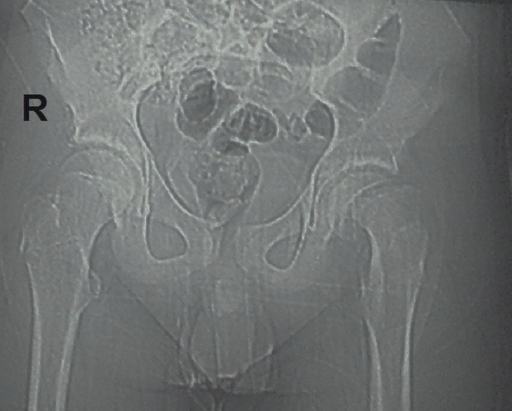
Anteroposterior hip graph of the patient showing increased left hip joint space compared to the right hip.

**Figure 2.**
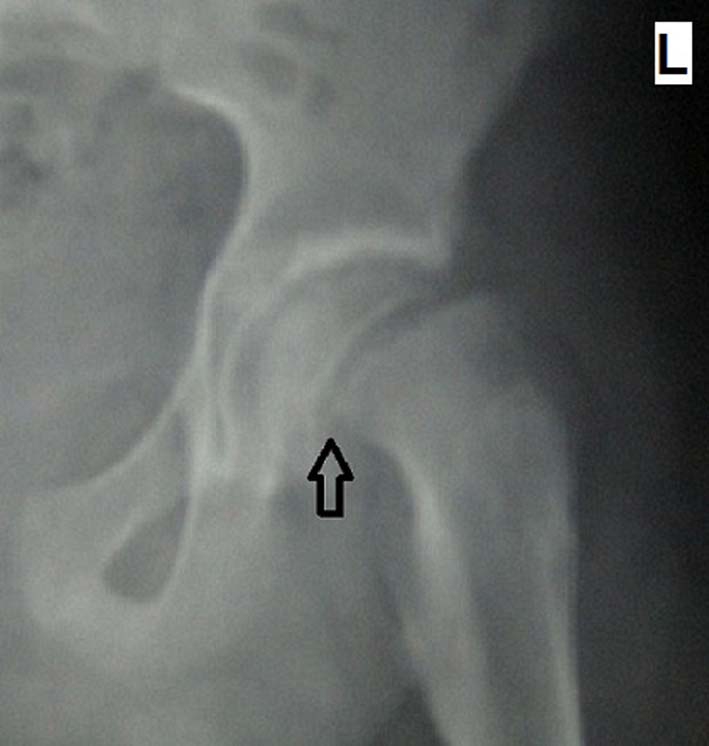
Frog-leg lateral left hip graph showing slippage of the capital femoral epiphysis on the proximal femoral metaphysis (black arrow).

A 16-year-old adolescent male presented to the emergency department with a chief complaint of left-sided hip pain of 3 weeks’ duration. The frequency of pain was intermittent and tended to be worst since the last 3 days, with the complaints of left knee pain and limping added on top of the pain. He had neither a history of trauma nor a previously known disease. His physical examination revealed an overweight adolescent with a body mass index of 26.6 kg/m^2^. He had a moderate antalgic gait with the left leg in external and internal rotation. He had also showed limited mobility of left hip in abduction, with no edema or crepitation and sensory or motor deficits. The examination of the contralateral hip was unremarkable. The anteroposterior hip radiograph showed increased left hip joint space compared to the right hip (Fig. 1). A frog-leg lateral left hip radiograph demonstrated dislocated capital femoral epiphysis on the proximal femoral metaphysis (Fig. 2). He was taken to surgery with the diagnosis of slipped capital femoral epiphysis and the right femoral epiphysis internally fixed with a single-cannulated screw under scopy guidance.

Slipped capital femoral epiphysis (SCFE), also named as adolescent coxa vara, is characterized by a growth disturbance of the proximal femoral growth plate, resulting in posterior and inferior displacement of the proximal femoral epiphysis (femoral head) on the metaphysis (femoral neck) [1, 2]. It is a common hip disorder which is known to be strongly related to overweight and obesity in adolescents and children aged 9 - 16 years [1, 3]. A retrospective review of emergency department records stated that 68% of children with SCFE had body mass index above the 95th percentile mean weight for age [2]. The most frequent presenting symptoms in patients with SCFE are pain in the affected hip, groin, thigh, or knee, limitation in hip range of motion, and often a gait disturbance (i.e., intermittent limp). The examination of contralateral hip should also be offered for evidence of symptoms [2, 4]. Anteroposterior and frog-leg (patient’s femur externally rotated) lateral radiographs are the appropriate radiographic views to be taken in patients with SCFE for diagnosis and treatment [1, 4]. The proper initial treatment for SCFE is in situ central single-screw fixation of the affected femoral epiphysis to the femoral metaphysis [4]. Although the potentially devastating complications of avascular necrosis and chondrolysis, the clinical outcome of children and adolescent patients with SCFE who are diagnosed early and surgically treated is generally good [5].

Early diagnosis and urgent surgery of SCFEs are important as these prevent the risk of further slippage of the femoral epiphysis and hip deformities. Thus, SCFE should always be considered in the differential diagnosis when a child or adolescent patient presents with complaints of limping knee, hip or thigh pain. To make a definitive diagnosis, appropriate radiographic views should be performed as in the present case.
